# High-efficient energy harvesting architecture for self-powered thermal-monitoring wireless sensor node based on a single thermoelectric generator

**DOI:** 10.1038/s41598-023-28378-6

**Published:** 2023-01-30

**Authors:** Albert Álvarez-Carulla, Albert Saiz-Vela, Manel Puig-Vidal, Jaime López-Sánchez, Jordi Colomer-Farrarons, Pere Ll. Miribel-Català

**Affiliations:** 1 Electronics and Biomedical Engineering Department, Universitat de Barcelona (UB), Marti i Franques, 1-11, 08028 Barcelona, Spain; 2 Industrial Engineering and Computer Science, Polytechnic School, University of Lleida (UdL), C. de Jaume II, 69, 25001 Lleida, Spain

**Keywords:** Electrical and electronic engineering, Energy infrastructure, Energy harvesting, Thermoelectric devices and materials

## Abstract

In recent years, research on transducers and system architectures for self-powered devices has gained attention for their direct impact on the Internet of Things in terms of cost, power consumption, and environmental impact. The concept of a wireless sensor node that uses a single thermoelectric generator as a power source and as a temperature gradient sensor in an efficient and controlled manner is investigated. The purpose of the device is to collect temperature gradient data in data centres to enable the application of thermal-aware server load management algorithms. By using a maximum power point tracking algorithm, the operating point of the thermoelectric generator is kept under control while using its power-temperature transfer function to measure the temperature gradient. In this way, a more accurate measurement of the temperature gradient is achieved while harvesting energy with maximum efficiency. The results show the operation of the system through its different phases as well as demonstrate its ability to efficiently harvest energy from a temperature gradient while measuring it. With this system architecture, temperature gradients can be measured with a maximum error of 0.14 $^{\circ }$C and an efficiency of over 92% for values above 13 $^{\circ }$C and a single transducer.

## Introduction

Phenomena such as the internet of things (IoT)^1^ or wireless sensor networks (WSNs)^2^ contemplate the installation of a number of sensors ranging from several hundreds to even thousands^2^. In these networks, the interest lies in powering the different sensor nodes autonomously. The most direct and widely solution is the use of batteries. But this has two main drawbacks: (1) an increase in the cost of operation and maintenance of the node, and (2) a significant environmental impact each time a battery is disposed. This, multiplied by hundreds of sensor nodes, makes the long-term sustainability of the network questionable. So powering the node locally by energy harvesting, enabling internet of battery-less things (IoBT)^3^, is a better alternative. To ensure the energy viability of these devices, two ways are used in the node: (1) reduce the number of components, and (2) reduce the computation. The first strategy is clear: reducing the number of components to be used means fewer components to be powered and, therefore, less consumption^4^. The second strategy consists of reducing the computation in the node^5^. However, reducing computation at the node in order to make its self-powering possible does not solve the problem, but rather moves it to another location. In this case, is moved it to data centres.

They see their activity increased due to an ever-increasing volume of data to be stored and an ever-increasing computational activity to process this huge amount of data which lead to an expected energy needs up to 1287 TWh in 2030^6^. The aim in these centres is to reduce the energy cost of their cooling or, in other words, to reduce the power usage effectiveness (PUE). PUE is a standard for measuring the efficiency of energy use in data centres defined by The Green Grid^7^ and is defined as:

1 $$\begin{aligned} \text {PUE} = \frac{\text {Total data center power}}{\text {IT equipment power}} \end{aligned}$$

In an ideal scenario, the PUE would be 1 and all the energy used in data centres would be used solely to power the servers. However, this is not the case and additional energy is required to power systems such as cooling, monitoring, lighting, etc. For example, Google data centres have a trailing 12-month PUE of 1.11 in 2020^8^, Microsoft a PUE of 1.125 in 2015^9^, Amazon a PUE of 1.2 reported in 2014^10^, and a survey in 2019 revealed that an average PUE for the other companies of 1.67^11^. With the goal of improving the current PUE in data centres, this paper presents a wireless sensor node that uses a thermoelectric generator (TEG) to collect the energy given off in the form of heat by a microcontroller unit (MCU) in a data centre. Simultaneously, using the same TEG, it monitors the temperature gradient between the MCU and its environment. This allows monitoring of the ambient temperature in the data center at a fine granularity that enables the implementation of thermal-aware server load management algorithms^12^. The deployment of a wireless battery-less sensor network and the implementation of thermal-aware server load management algorithms are two pathways that enable improving the PUE. The data are sent wirelessly with LoRaWAN while the power is extracted from the TEG efficiently using a maximum power point tracking (MPPT) algorithm.

The main lines of research focused on the use of TEGs as a clean power source are the development and fabrication of the TEGs themselves, the design of power management units (PMUs) for efficient energy extraction, and the design and development of systems and architectures that use TEGs to perform continuous monitoring of a parameter of interest. For example, a new assembly technology is presented that allows to increase the integration density of TEGs^13^. Intended for wearable self-powered devices, a TEG fabricated using this technology allows achieving higher power values, up to 91 μW for a temperature gradient of 5 $^{\circ }$C, than other comparable TEGs. On the other hand, a TEG-based energy harvesting system for powering wireless sensor nodes is presented^14^. The system consists of two boost converters and is notable for allowing the system to be started with a voltage of up to 62 mV. All this, while achieving an efficiency of between 44.2 and 75.4%. Another interesting work presents a self-powered sensor node for monitoring a gas turbine^15^. The device is capable of delivering a regulated voltage of 2.4 V and a maximum power of 0.92 W for a turbine temperature of 325 $^{\circ }$C. And a new MPPT algorithm is presented to efficiently extract energy from TEG^16^ . The presented method stands out for its simplicity by requiring very few additional components, which also allows to reduce the power consumption of its implementation. With this method, the TEG is able to work at an operating point deviated by no more than 1.87% with respect to its maximum power point (MPP).

However, there is not much research on the use of TEG as a power source and sensor simultaneously. This is also stated in Shi et al. where the authors present one of the few existing works in this regard^17^. A TEG-based self-powered wireless sensor node is presented in which the TEG is used as a power source and sensor. The node consists of a TEG connected to an electronic reader consisting of a PMU, an analog-to-digital converter (ADC), a microcontroller and a radio frequency (RF) transmitter. The output voltage of the TEG is used as an indicator signal of the temperature gradient to which it is subjected. This signal is captured and digitized by the ADC and transmitted wirelessly to a host. However, this approach suffers from a drawback that the solution presented in this work solves. This drawback is that the operating point of the TEG is not controlled. This has two consequences. The first is that the output voltage of the TEG cannot be used as a direct indicator of the temperature gradient to which it is subjected. It could be used if the TEG is in open circuit, but this is not the case. Although the authors indicate that the impedance of the ADC is high, it should be noted that the output of the TEG is also connected to the PMU that is responsible for powering the entire system. The average consumption of the device, which is not indicated, makes that the TEG is not in an open-circuit situation. In addition, the dynamic consumption causes the operating point of the TEG to fluctuate uncontrollably. This causes an error in the measurement of the temperature gradient, in this case, of 0.5 $^{\circ }$C. The second consequence is that energy is not extracted from the TEG efficiently. The TEG needs to operate with a load impedance equal to its internal impedance in order to deliver maximum power. The use of MPPT algorithms is a must when operating with low temperature gradients or when the complexity of the system (i.e., its power consumption) is notorious. No efficiency value is given concerning energy extraction from the TEG. Finally, the work indicates a current consumption of 17 mA of the communication module, but does not describe its design and implementation being a key module of the system.

Another very interesting solution to extract energy and measure temperature using a thermoelectric generator is presented by Wen et al.^18^. In this approach two TEGs are used following a double-chain configuration. One has the role to generate energy and the second one as a sensor instead of our proposal where just one thermoelectric module is used for both roles at once. The authors use the harvested energy to power a calculator by generating a regulated voltage of 3.3 V to demonstrate the device’s ability to power commercial solutions. Furthermore, for the measurement of the power extracted from the TEG, which is 2.9 μW at a temperature gradient of 50 $^{\circ }$C and a load resistor of 1.8 k$\Omega$, the load impedance is set manually without any autonomous matching. This means that in the event of variations in the equivalent input impedance of the system powered by the TEG, energy is not extracted from the latter efficiently which is other advantage of our system. Finally, it is not shown whether the solution is able to power a device to enable wireless transmissions, which is one of the main desirable features of such devices. A calculator is powered which does nothing with the measurement performed by the solution. In other words, the wearable multi-sensing double-chain thermoelectric generator is able to collect energy and measure temperature, but it is not able to output the measurement, which makes it far from being a viable commercial solution in the short term.

While the presented solution has a great potential in all applications where continuous monitoring of temperature gradients is required and where it is not feasible to make use of batteries or connections to the power grid, such as applications in structural health monitoring^19,20^, temperature gradient monitorization in smart buildings for the generation of energy efficiency models^21,22^, or non-invasive measurement of the temperature of fluids in pipelines using TEGs^23^, this work presents an approach in which a TEG is operated as a power supply and sensor simultaneously while extracting energy from the TEG with maximum efficiency in the framework of an application for monitoring the temperature gradient between a server microprocessor and the environment. That is, at its MPP. For this purpose it controls the operating point of the TEG by using an MPPT algorithm. Also described is the supercapacitor-based, power-aware wireless transmission management algorithm used to enable wireless transmission of data via LoRa/LoRaWAN.

## Results

### The thermoelectric as a generator and sensor

A TEG is a solid state device that transduces heat energy into electrical energy. As result, an open-circuit voltage (OCV) is originated across the ends of the thermocouple, and a current flow is originated from one end to the other when an electrical load is connected. The OCV, for a given temperature gradient, is expressed by:

2 $$\begin{aligned} V_{TEG_{OCV}} = S\Delta T, \end{aligned}$$

where *S* is the Seebeck coefficient and $\Delta T$ the temperature gradient across the TEG. Meanwhile, when a load is attached to the TEG terminals, the TEG output voltage, which depends on its internal resistance $R_{TEG}$, is:

3 $$\begin{aligned} V_{TEG} = \frac{S\Delta T}{\frac{R_{TEG}}{R_L}+1}, \end{aligned}$$

where $R_L$ is the electrical load. Similarly, the TEG output current can be expressed as:

4 $$\begin{aligned} I_{TEG} = \frac{S\Delta T}{R_{TEG}+R_L} \end{aligned}$$

**Figure 1:**
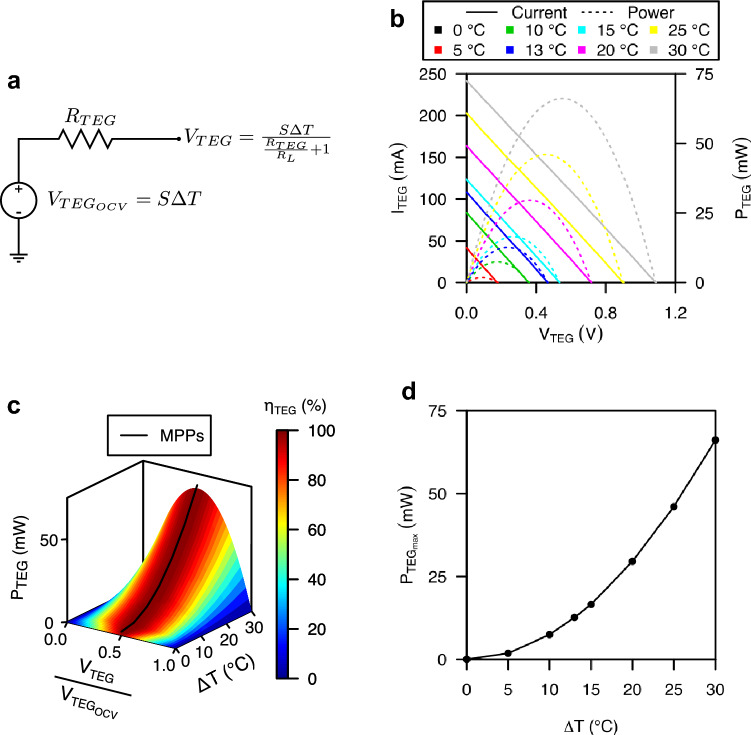
TGM-127-1.0-2.5 thermoelectric generator experimental characterization. (**a**) Simplified lumped electrical model of a general-purpose thermoelectric generator. (**b**) Thermoelectric generator’s polarization curves for different temperature gradients ($\Delta T$). (**c**) 3D plot of the power outputted by the thermoelectric generator ($P_{TEG}$), its efficiency ($\eta _{TEG}$) and the maximum power points versus its polarization voltage ($V_{TEG}$) normalized respect its open-circuit voltage ($V_{TEG_{OCV}}$), and the temperature gradient ($\Delta T$). (**d**) Transfer function of temperature gradient ($\Delta T$) to power at maximum power point ($P_{TEG_{max}}$).

Derived from Eqs. (2)–(4), Fig. 1a shows the simplest lumped electrical model of a TEG^24^. The measured voltage vs. current polarization curves of a TGM-127-1.0-2.5 TEG are shown in Fig. 1b. The temperature gradient range used has been selected taking into account the American Society of Heating, Refrigerating and Air-Conditioning Engineers (ASHRAE) ambient temperature guidelines and the operating temperatures of dedicated microprocessors for servers. The minimum temperature gradient is the critical parameter to validate the feasibility of the solution. ASHRAE recommends ambient temperatures from 18 to 32 $^{\circ }$C, which is the most restrictive case and corresponds to the recommendations for type A1 installations^25^. As an example of operating temperatures of dedicated microprocessors for servers, we have taken as a reference a 3rd Gen AMD EPYC processor used by the main companies that own data centres^26^. Benchmarking temperature data has been used to establish a minimum operating temperature of 45 $^{\circ }$C^27^ and the manufacturer’s specifications to establish a maximum operating temperature of 81 $^{\circ }$C^28^. These temperatures set a minimum temperature gradient of 13 $^{\circ }$C. In this work, we have characterized the TEG for a temperature gradient range from 0 $^{\circ }$C to the maximum temperature gradient that the used test platform is capable, which is 30 $^{\circ }$C, with a temperature gradient step of 5 $^{\circ }$C. The TEG has a Seebeck coefficient and an output resistance of 36 mV $^{\circ }$C$^{-1}$ and 4.4 $\Omega$, respectively. For the minimum temperature gradient of 13 $^{\circ }$C, the TEG is capable of generating an OCV of 465 mV, a short-circuit current (SSC) of 108 mA, and a maximum power at the MPP of 12.7 mW. The performance of a TEG as power source can be evaluated extracting power versus voltage curves for different temperature gradients. From these curves, we can extract characteristics such as the maximum power for a given temperature gradient, or, more importantly, the operating point in which this maximum power is extracted. We indicate how efficiently the TEG is being operated by means of the parameter $\eta _{TEG}$, defined as:

5 $$\begin{aligned} \eta _{TEG} = \frac{P_{TEG}}{P_{TEG_{max}}}, \end{aligned}$$

where $P_{TEG}$ is the power extracted from the TEG for a given operating point and $P_{TEG_{max}}$ is the maximum power that can be extracted at MPP. If the operating voltage of the TEG $V_{TEG}$ is normalized with respect the corresponding OCV, as shown in Fig. 1c, we can see how the maximum power, i.e., a $\eta _{TEG}$ efficiency of 100%, is achieved for a polarization voltage equal to one half of the OCV for the same temperature gradient. Usually, from polarization curves, a voltage vs. temperature gradient curve for a fixed load current is extracted to evaluate the performance of a TEG as sensor. When operated as a sensor, we set the operating point of the TEG in order to maximize its characteristics as a sensor. For example, to maximize its sensitivity when the voltage is used as output signal, we set the TEG under an open-circuit operating point. However, when using current as the output signal, we set the TEG under a short-circuit operating point in order to get maximum sensitivity. In these two operating points, no power is extracted from the TEG, making unfeasible the usage of the TEG as a power source. With the TEG as a power source, the MPP must be found using MPPT algorithms.

Finally, Fig. 1d shows the maximum power at MPP generated by the TEG for each temperature gradient. The maximum power shows a dependence on the temperature gradient which is expressed by the function

6 $$\begin{aligned} \begin{aligned} P_{TEG_{max}}&= {74.83}\frac{\upmu \text {W}}{\text {C}^{2}}\Delta T^2+{76.37}\frac{\upmu \text {W}}{{}^{\circ }\text {C}}\Delta T\\ R^2&= {0.9998} \end{aligned} \end{aligned}$$

which is used by the back-end module to compute the measured temperature gradient.

#### Maximum power point tracking algorithms and the maximum power to temperature gradient transfer function

Several MPPT algorithms exist to track the MPP of a TEG for given operating conditions. But all seek the same goal: to apply to the TEG a load impedance that matches its internal resistance. From Eqs. (3)–(4), the condition to reach the MPP is:

7 $$\begin{aligned} \frac{\partial P_{TEG}}{\partial R_L}= & {} \frac{\partial }{\partial R_L} \left( \frac{(S\Delta T)^2}{\frac{R_{TEG}^2}{R_L}+2R_{TEG}+R_L} \right) \end{aligned}$$

8 $$\begin{aligned} \frac{\partial P_{TEG}}{\partial R_L}= & {} 0 \longleftrightarrow R_L=R_{TEG} \end{aligned}$$

that is equivalent to apply a load voltage equal to one half of its OCV. Thus, one of the most common and well-known MPPT algorithms is the fractional open-circuit voltage (FOCV) MPPT algorithm^29^. The TEG is periodically disconnected from the system to set it in open-circuit condition and sample its OCV. Then, the TEG is reconnected to the system and its operating voltage $V_{TEG}$ is regulated to one half of its OCV by means of the applied load impedance. This algorithm presents one major drawback: no power is extracted when the TEG is disconnected from the system to measure its OCV. Thus, the maximum efficiency $\eta _{TEG}$ is achieved while the TEG is connected and regulated to its MPP, but the average efficiency $\overline{\eta _{TEG}}$ decreases because of the power losses introduced into the system during the sampling periods incurring less energy extraction. Furthermore, if the sampling frequency is reduced to increase $\overline{\eta _{TEG}}$, this can lead to an even lower $\overline{\eta _{TEG}}$ because the algorithm losses its capability to track the variation of the MPP due to variations of the temperature gradient during the long no sampling periods. Another MPPT algorithm is the perturb and observe (P &O)^30^. In this algorithm, as its name states, the TEG output voltage (or current, analogously) is slightly perturbed incrementing or decrementing its value while the resulting TEG output power is observed. If the TEG output power increases, the next perturbation of the TEG output voltage (or current) will be in the same direction. Otherwise, if the TEG output power decreases, the next perturbation will be in the opposite direction. Resulting from the P &O algorithm, the TEG operating point will reach its MPP and will oscillate around it. This oscillation of the TEG operating point around MPP leads to a high but not to a theoretically perfect $\eta _{TEG}$. This oscillation can be reduced decreasing the perturbation step at expense of a slower tracking speed. As benefit, in the P &O algorithm, there is no need to disconnect the TEG from the system to track its OCV or SSC. Thus, a higher $\overline{\eta _{TEG}}$ is achieved.

While the P &O algorithm enables the tracking of the MPP without disconnecting the TEG, the algorithm can also be used for a completely different purpose: to measure the temperature gradient across the TEG. A temperature gradient versus maximum power ($\Delta T$ − $P_{TEG_{max}}$) transfer function can be obtained from the polarization curves. As the P &O algorithm tracks the MPP, the temperature gradient across the TEG can be obtained from the measurement of the TEG output power, which is already monitored by the algorithm itself. Thus, only one single TEG is needed to harvest energy from the environment and to simultaneously sense a temperature gradient; meanwhile, the former is done efficiently. The presented solution makes use of the P &O algorithm to efficiently extract energy from the TEG while simultaneously using it as a temperature gradient sensor.

### The self-powered electronic module

**Figure 2:**
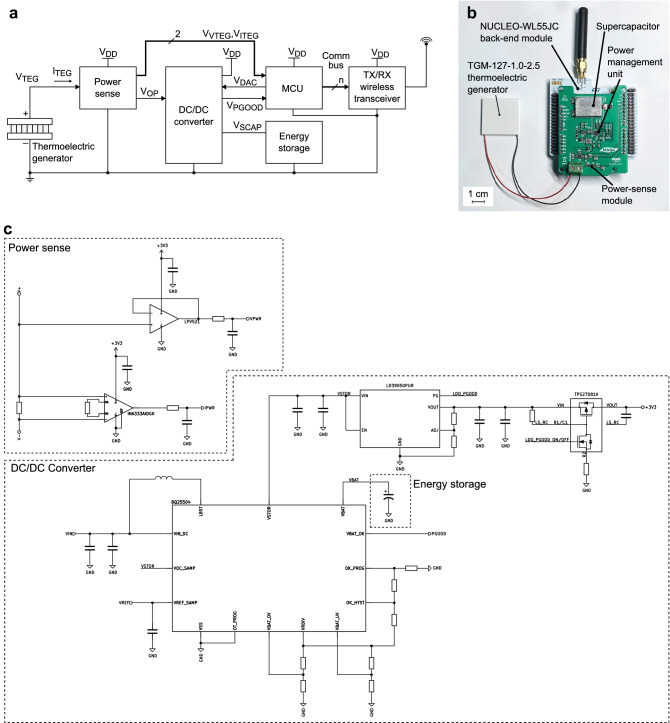
The self-powered thermal-monitoring wireless sensor node prototype based on a single thermoelectric generator. (**a**) Block diagram of the system architecture. (**b**) Photograph of device implementation. (**c**) Circuit diagram of the implemented prototype.

Common approach for TEG-based self-powered devices has three main modules: (1) a PMU, (2) a front-end and (3) back-end modules. The first one is responsible for the power extraction from the TEG. Due to the usually low voltage level outputted by TEGs^31^, the main task of the PMU is to boost the input voltage to generate a regulated voltage supply able to power the device. Normally present in these devices, an auxiliary task is to regulate its equivalent input impedance by means of a MPPT algorithm to maximize $\eta _{TEG}$. This module can be supported with an energy storage module that stores the surplus harvested energy providing a longer autonomy to the device or the capability to attend a punctual high power requirement. The front-end module is responsible to interface the sensor used for the temperature gradient measurement, which can be the TEG or an additional sensor. When the temperature gradient is measured using the TEG itself, one option is to use the disconnection period during the MPPT algorithm execution to measure the OCV. This implies a lower $\overline{\eta _{TEG}}$ due to the power losses introduced into the system during TEG disconnections. The last module, the back end, is application dependent, but usually it consists of a MCU, responsible for data processing and the control of the MPPT algorithm execution; and an interface to output the measurement, such as a wireless transmitter or a display. The block diagram and a picture of the system presented in this work are shown in Fig. 2. The device uses the P &O algorithm and modulates the equivalent input impedance of its PMU to maximize $\eta _{TEG}$. While power is being extracted from the TEG, the front-end or power-sense module, placed in the current path between the TEG and the PMU, senses the power outputted by the former. This allows to simultaneously measure the temperature gradient across the TEG. The front-end module outputs two voltage signals $V_{VTEG}$ and $V_{ITEG}$ as indicators of the voltage and current levels outputted by the TEG, respectively. These two signals are connected to a MCU that samples them with its analog-to-digital converter (ADC) and computes the TEG output power $P_{TEG}$. With $P_{TEG}$ and $V_{VTEG}$, the MCU controls the execution of the P &O algorithm and, using its digital-to-analog converter (DAC), generates an analog control signal $V_{DAC}$ that is connected to the PMU closing the feedback loop and controlling its equivalent input impedance. In addition, the MCU converts the measured TEG output power to temperature gradient and sends it to a wireless transceiver to transmit the data to a gateway or host. A low-power wide-area network (LPWAN) transmission, like in long-range modulation wide-area network (LoRaWAN), can require a relatively high-current consumption of 17 mA as minimum^32^. If the TEG is not able to meet the required power consumption at the moment of the transmission, the latter will be unfeasible or, directly, the entire system will be shutdown. To address this, an energy storage module is included with the PMU. The system harvests energy through the TEG and stores it in the energy storage module. Once enough energy is stored, the PMU rises the signal $V_{PGOOD}$ indicating to the back-end module that the wireless transmission is energetically feasible and can be done. Once the energy level in the storage module drops, the signal $V_{PGOOD}$ goes down until enough energy is harvested again. In energy storage modules based on a supercapacitor, the polarization voltage $V_{SCAP}$ is used as an indicator of the energy stored defined as:

9 $$\begin{aligned} E_{SCAP} = \frac{1}{2}C_{SCAP}V^2_{SCAP}, \end{aligned}$$

where $V_{SCAP}$ is the voltage across the supercapacitor and $C_{SCAP}$ its capacitance.

The choice of the supercapacitor capacitance is a crucial aspect in this type of device because it has a direct impact on the initial time needed to charge the supercapacitor and to be able to perform the first wireless communication. In turn, capacitance also influences the voltage drop caused by a decrease in stored energy. In order to facilitate system start-up and minimize the time required to perform the first wireless communication, the capacitance has been sized to be as small as possible to meet the energy requirements of the wireless communication. Following Eq. (9), for the same amount of energy a smaller capacitance can be obtained if a larger voltage drop is allowed. On the other hand, a higher capacitance value would allow a lower voltage drop but would also lead to a longer start-up time.

### Back-end module consumption

**Figure 3:**
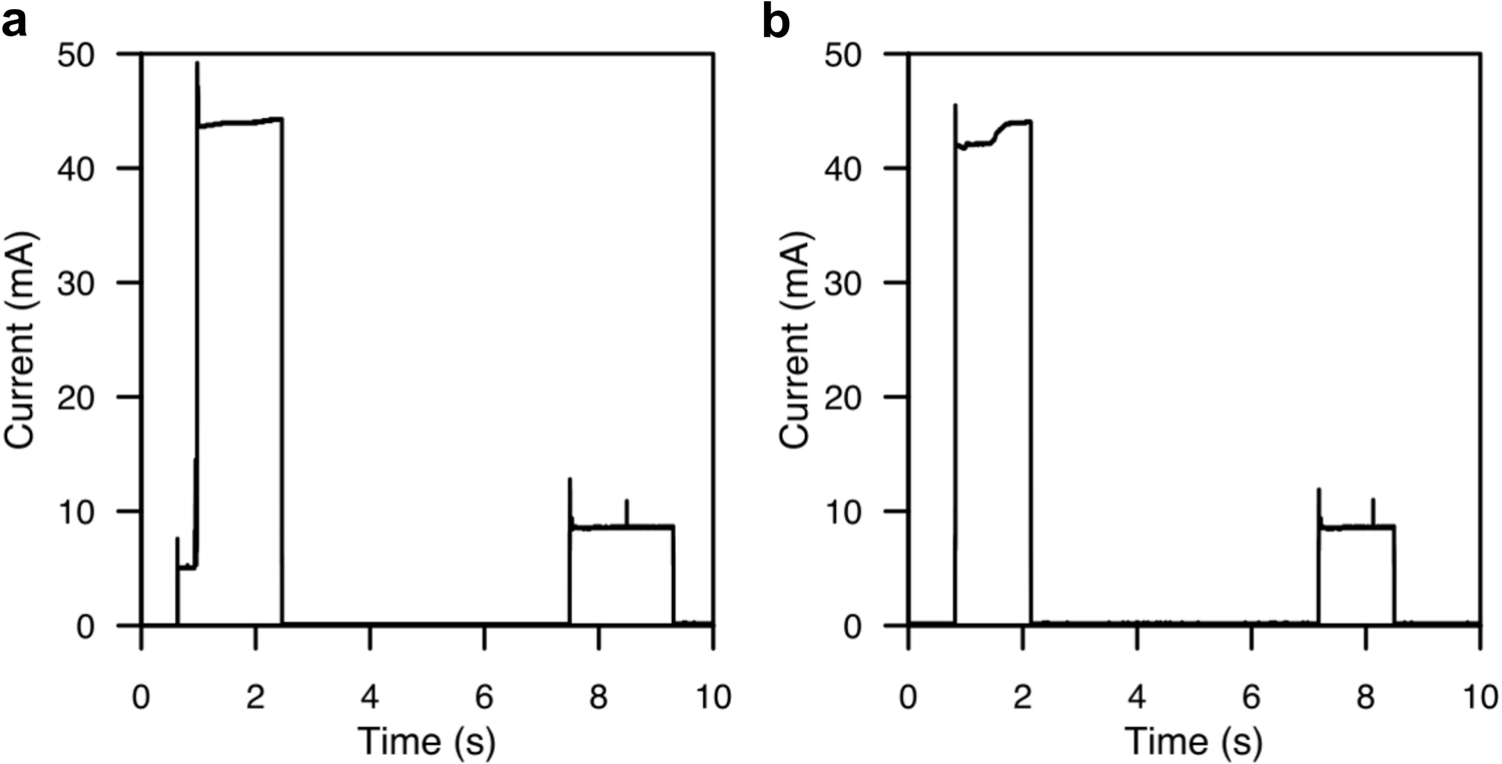
Current consumption waveforms of the back-end module. (**a**) During microcontroller’s initialization and LoRaWAN over-the-air activation. (**b**) During a LoRaWAN transmission.

Figure 3 shows the current consumption waveforms for both join procedure and data transmission. To join the LoRaWAN, the back end needs a total energy of 148.9 mJ, while it needs a total energy of 122.4 mJ to transmit data. In terms of current consumption, the back end consumes 42.6 mA average current consumption during transmission, and 8.6 mA during reception. During idle, when the MCU is in ultra-low-power operation mode (LPM), the average current consumption is 3.7 μA. Using the most restrictive case of 148.9 mJ, a supercapacitor greater than 27.4 mF is required to ensure a maximum voltage drop at the supercapacitor below 3.3V.

### Device start-up and operation

**Figure 4:**
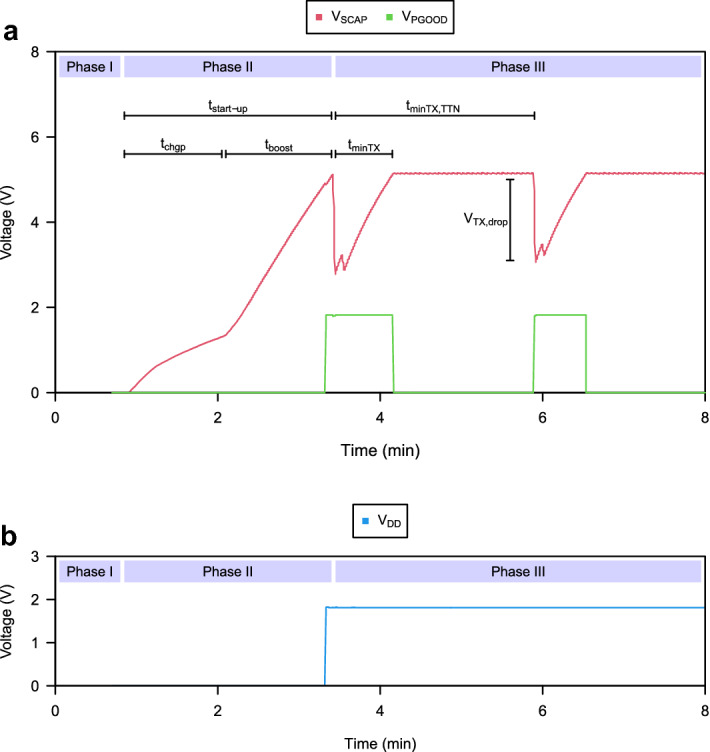
Transient waveforms during the start-up and steady-state operation of the device for a temperature gradient of 13 $^{\circ }$C. (**a**) The voltage across the supercapacitor ($V_{SCAP}$), and the signal indicator of the availability of enough energy to perform a transmission ($V_{PGOOD}$). (**b**) The regulated voltage supply ($V_{DD}$).

**Figure 5:**
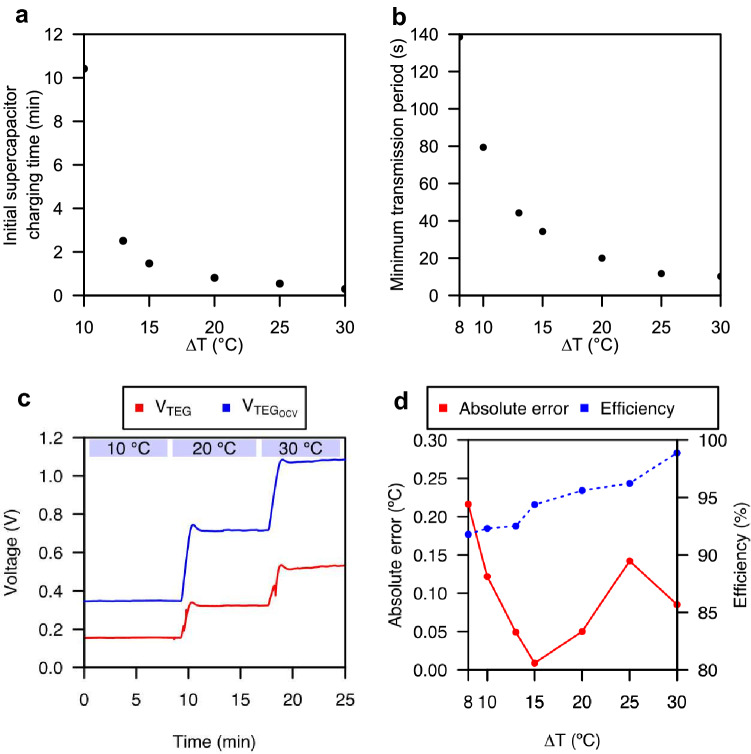
Characterization results of the device. (**a**) Time required by the device to charge the initially discharged supercapacitor to 5V to enable wireless communication. (**b**) Time period between transmissions versus temperature gradient. (**c**) Thermoelectric generator’s operating ($V_{TEG}$) and open-circuit ($V_{TEG_{OCV}}$) voltages for different temperature gradients ($\Delta T$). (**d**) Absolute error of the temperature gradient measurement and efficiency power extraction $\eta _{TEG}$.

With the supercapacitor fully discharged, when connected to the TEG with a temperature gradient applied, the PMU starts to extract power from the TEG and three phases are easily distinguishable. Shown in Fig. 4, phase I corresponds to the period in which the TEG has not yet been connected to the device and no energy is being extracted from it. Phase II starts as soon as the TEG is connected to the device. This is when the PMU starts extracting power from the TEG through its charge pump firstly—during $t_{chgp}$—and its boost converter—during $t_{boost}$ —once a boosted voltage of 1.8V is reached. When $V_{SCAP}$ reaches a voltage of 5V, the MCU turns on applying the P &O algorithm, and joins the LoRaWAN network. Due to the OTAA, the energy in the supercapacitor decreases and $V_{PGOOD}$ goes high. Once joined, phase III starts and the temperature gradient is sent every time enough energy is stored in the supercapacitor. Both $t_{tminTX,TTN}$ and $t_{minTX}$ are shown in the Fig. 4. The former corresponds to the minimum period of time that must elapse between transmissions to comply with TheThings Network’s fair use policy^33^. The latter corresponds to the actual minimum transmission period and is only conditioned by the availability of energy in the supercapacitor. From the experiments performed for different temperature gradients, we have measured the time required to charge the supercapacitor, initially discharged, to 5V and enable wireless communication, denoted as $t_{start-up}$, and the time required to charge the supercapacitor between transmissions, denoted as $t_{minTX}$ as it sets the minimum transmission period. $t_{start-up}$ and $t_{minTX}$ have been measured and are shown in Fig. 5a–b. The solution presents a start-up time that shows a dependence on the temperature gradient as could be expected. Higher temperature gradients allow the TEG to increase the delivered power and speed up the charging of the supercapacitor. For a minimum temperature gradient of 10 $^{\circ }$C, the supercapacitance takes 625 $^{\circ }$C to charge, while for a maximum temperature gradient of 30 $^{\circ }$C, the supercapacitance requires 18 s. The critical start-up time for the minimum temperature gradient of 13 $^{\circ }$C is 2.5 min, while for temperature gradients below 10 $^{\circ }$C the system is not able to start. This operation limit is due to the minimum cold-start input voltage required by the BQ25504 and the low Seebeck coefficient of the TEG. The transmission period shows the same dependence on the temperature gradient. In this case, for the critical temperature gradient of 13 $^{\circ }$C, a transmission period of 44s has been measured. On the other hand, it has been possible to observe that the device can transmit with temperature gradients above 8 $^{\circ }$C. In this case, the temperature gradient is less than the temperature gradient required to start the system. This is because the BQ25504, once started, requires a lower voltage level. An important aspect of the solution is its ability to follow the temperature gradient to extract energy from the TEG efficiently while being able to obtain the temperature gradient to which the TEG is subjected. This can be seen in Fig. 5c. It can be seen how the prototype is able to track temperature gradient changes by controlling the TEG operating voltage to one half of its OCV. As a result, efficiencies from 91.8 to 98.8% are achieved, getting an efficiency of 92.5% for the critical temperature gradient of 13 $^{\circ }$C. In addition, it can be seen how the device is able to calculate the temperature gradient and transmit it wirelessly with absolute error below 0.22 $^{\circ }$C, as shown in Fig. 5d. Lower $\eta _{TEG}$ efficiencies are shown for lower temperature gradients. This is because while the TEG operating voltages decrease with applied temperature gradients, the step in voltage used in the P &O algorithm is constant causing there to be a larger relative oscillation around the MPP for lower temperature gradients. The device performs its operation with a power consumption of 3.256 mW between transmissions.

Summarising the results obtained, the solution presented is capable of operating from a minimum temperature gradient of 8 $^{\circ }$C, a minimum gradient of 10 $^{\circ }$C being necessary during start-up. For the minimum temperature gradient, the system is able to start up between 18 and 625s. Using the P &O algorithm, the device is able to extract energy from the TEG with an efficiency of between 91.8 and 98.9% while simultaneously measuring the temperature gradient with a maximum error of 0.22 $^{\circ }$C. With a power consumption of 3.256 mW, the sensor node is capable of transmitting data wirelessly via LoRaWAN (868 MHz) with a power of 14 dBm to reach distances of up to 10 km line-of-sight distance. Table 1 shows a summary of the obtained results compared with other state-of-the-art solutions considered by the authors to be some of the most relevant ones.

**Table 1:** Summary of the obtained results versus other state-of-the-art solutions.

Parameter	Xia et al.^34^	Brunelli et al.^35^	Wen et al^18^	Shi et al.^17^	This work
Operating temperature gradient range	n/a	10–40 $^{\circ }$C	50–n/a $^{\circ }$C	23–n/a $^{\circ }$C	8–30 $^{\circ }$C
Absolute error	n/a	n/a	n/a	n/a–0.5 $^{\circ }$C	0.01–0.22 $^{\circ }$C
Maximum power point tracking algorithm	FOCV	None	None	None	P &O
Efficiency $\eta _{TEG}$	19–81%	n/a	n/a	n/a	91.8–98.9%
Power consumption	34.6 μW	6.5 μW	n/a	n/a	3.256 mW
Start-up time range	n/a	n/a	19.6–369.0 s	37 s	18–625 s
Data transmission period range	65 s	0–110 s	None	n/a	10–139 s
Communication protocol	ZigBee	Simplicity	None	Gazell	LoRaWAN
Base frequency transmission	2.4 GHz	2.4 GHz	None	2.4 GHz	868 MHz
Transmission power	n/a	n/a	None	n/a	14 dBm
Maximum transmission range	${<\;100}$m	${<\;100}$ m	None	${<\;100}$ m	${<\;10}$ km

## Discussion

A novel approach to measure temperature gradients using a single TEG that, in turn, efficiently powers the system to enable the development of self-powered wireless sensor nodes has been presented. The prototype presented to validate this approach, is intended to measure the temperature gradients in data centres to apply thermal-aware server load management algorithms. The oscillation around the MPP is intrinsic to the used P &O algorithm and results in a gradient temperature measurement error of up to 0.22 $^{\circ }$C for a temperature gradient of 8 $^{\circ }$C, and an efficiency of 92.5% for a temperature gradient of 13 $^{\circ }$C. This is because the voltage step of the algorithm is constant. A dynamic voltage step would help to improve these specifications for low temperature gradients. Even so, the system is able to operate with a maximum error of 0.14 $^{\circ }$C for a temperature gradient of 25 $^{\circ }$C, and an efficiency of up to 98.8% for a temperature gradient of 30 $^{\circ }$C. The node is able to perform a wireless transmission using LoRaWAN for a minimum temperature gradient of 8 $^{\circ }$C with a minimum transmission period of 44s for the critical gradient of 13 $^{\circ }$C. On the other hand, a minimum gradient of 10 $^{\circ }$C is required to be able to start the system. All this is achieved with a power consumption of 3.256 mW between transmissions. There are aspects to improve that are part of our future research work. While it is not critical for this application, one aspect to improve is reducing the minimum system start-up voltage by replacing the currently used commercial integrated circuit (IC) with a custom integrated version that requires a lower voltage. The second one aspect to work on is the P &O algorithm so that it applies a dynamic voltage step to help improve the error in the measurement of the temperature gradient and the efficiencies obtained. With all this, the presented solution enables the development of self-powered wireless sensor nodes for monitoring temperature gradients by using a single TEG operated in an energetically efficient way.

## Methods

We have implemented a prototype using current available commercial-off-the-shelf (COTS) discrete components. Then, we have validated its operation under controlled conditions with a commercial TEG. We describe the materials and methods used as follows.

### Device design and fabrication

We have used a TGM-127-1.0-2.5 from Kryotherm. We have implemented the PMU, the energy storage, and the power-sense modules on a 53mm $\times$ 70mm double-sided printed circuit board (PCB). A NUCLEO-WL55JC evaluation board from STMicroelectronics has been used to implement the back-end module. The PMU is based on a BQ25504 IC from Texas Instruments. We have disconnected the BQ25504’s open-circuit voltage sampling input and have connected a controlled voltage to the IC’s voltage reference input to regulate the TEG’s output voltage to a desired/controlled voltage value. The harvested energy is stored on a 30mF supercapacitor from KYOCERA AVX. The supercapacitor value has been selected taking into account the energy required to perform a wireless transmission and the maximum voltage drop allowed in the supercapacitor during a period of high energy demand. When enough energy is harvested to perform a wireless transmission, the PMU turns low the $V_{PGOOD}$ signal that is connected to the MCU. Finally, the PMU has a second dc-dc converter, a LD39050 from STMicroelectronics, is used to generate a 1.8V regulated voltage supply $V_{DD}$. For the power-sense module, we have used an INA333 instrumentation amplifier (InAmp) and a LPV521 operational amplifier (OpAmp) from Texas Instruments to measure the output current of the TEG $I_{TEG}$ via a shunt resistor of 0.2 $\Omega$ and the TEG output voltage $V_{TEG}$, respectively. They are routed to different input channels of the MCU’s ADC for conversion and subsequent calculation of the TEG output power $P_{TEG}$ and the corresponding temperature gradient $\Delta T$. The back-end module is based on a NUCLEO-WL55JC evaluation board from STMicroelectronics. The module has a 32-bit dual-core STM32WL55JC MCU with essential features and peripherals for the application like an LPM, a 12-bit ADC with multiple input channels, a 12-bit DAC to generate the control signal for the input impedance of the PMU, and a RF transmitter with LoRa. Once the MCU is powered to its minimum operating voltage, it initializes all its peripherals and proceeds to join a LoRaWAN using the over-the-air activation (OTAA) method. Once it has joined the network, the MCU enters in LPM. From then on, the MCU wakes up and samples $V_{VTEG}$ and ${V_{ITEG}}$ to calculate the power outputted by the TEG and applies the P &O algorithm. The temperature gradient to which the TEG is subjected is also calculated using Eq. (6) previously obtained from the TEG characterization. Upon completion of the algorithm iteration, the MCU returns to LPM. In turn, the MCU has an interrupt configured to know when there is enough power in the supercapacitor to perform a wireless transmission. This is indicated by the $V_{PGOOD}$ signal from the PMU.

### Thermoelectric generator and device characterization

A linear voltage sweep measurement has been carried out on the TEG using a B2962A source meter unit (SMU) from Keysight for different temperature gradients. To set the temperature gradients, a custom platform based on two peltier cells facing each other was used. The temperature gradient range used has been from 0 to 30 $^{\circ }$C, with a temperature gradient step of 5 $^{\circ }$C. The TEG has been also characterized for the critical temperature gradient of 13 $^{\circ }$C. In order to select the proper supercapacitor value, the current consumption of the back-end module has been measured using the B2962A SMU during the two most energy-demand processes: the OTAA and the wireless transmission. To study the start-up and steady state operation of the prototype, the voltage at the supercapacitor $V_{CAP}$, the regulated voltage $V_{DD}$, and the signal $V_{PGOOD}$ are sampled using an InfiniiVision 3000A oscilloscope from Keysight. For the measurements, the device with its supercapacitor fully discharged is connected to the TEG subjected to a temperature gradient. From these measurements, the start-up times $t_{start-up}$ and the minimum transmission periods $t_{minTX}$ for each temperature gradient are extracted. The former corresponds to the time from the connection of the TEG until connection to the LoRaWAN. The latter corresponds to the time between transmissions during steady-state operation. To validate the capability of the device to track the temperature gradient and extract power from the TEG efficiently, the TEG operating voltage $V_{TEG}$ and its OCV $V_{TEG_{OCV}}$ have been sampled for different temperature gradients. $V_{TEG_{OCV}}$ has been measured using a second TGM-127-1.0-2.5 TEG thermally connected in parallel and under open-circuit condition. The B2962A SMU has been used to measure the power consumption of the device. Finally, to evaluate the accuracy of the system, absolute error have been measured using the same temperature gradient range that for TEG characterization. The efficiency $\eta _{TEG}$ has been also measured along the temperature gradient range.

## Data Availability

The datasets generated during and/or analysed during the current study are available from the corresponding author on reasonable request.
